# Adenosine to inosine mRNA editing in fungi and how it may relate to fungal pathogenesis

**DOI:** 10.1371/journal.ppat.1007231

**Published:** 2018-09-27

**Authors:** Ines Teichert

**Affiliations:** Lehrstuhl für Allgemeine und Molekulare Botanik, Ruhr-Universitaet Bochum, Germany; University at Buffalo School of Medicine and Biomedical Sciences, Buffalo, UNITED STATES

One central hypothesis of molecular biology is that a protein sequence can be deduced from the DNA sequence. However, diverse processes at the DNA, mRNA, and even protein level can lead to protein sequences that differ from the deduced sequence. One such process is co- or post-transcriptional RNA editing. RNA editing is found in all domains of life and in diverse RNA species from bacteria and archaea as well as plastids, mitochondria, and nuclei of eukaryotes [1,2,3,4]. This review will give an overview of different types of mRNA editing and then focus on fungal mRNA editing, which was described only recently.

## Q1: What is mRNA editing?

**Fig 1:**
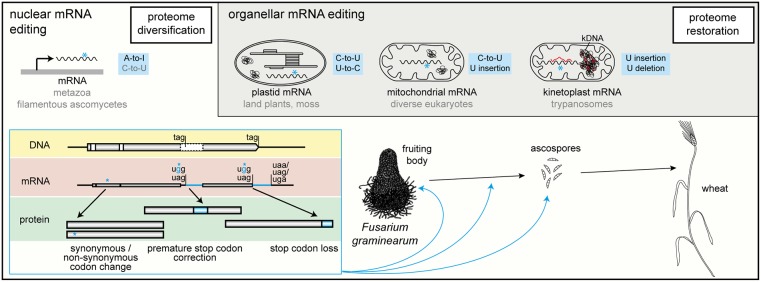
Types of mRNA editing and possible relation to fungal pathogenesis. Editing of nuclear mRNA leads to diversification of the proteome, whereas editing of plastid, mitochondrial, and kinetoplast mRNA is mostly restorative. Distinct editing events (marked by blue asterisks) occur in organellar as well as nuclear transcripts. Undulated lines indicate transcripts. In the human parasite *Trypanosoma brucei*, transcripts derived from maxicircle DNA (black in kDNA) are edited using guide RNA (red) derived from minicircle DNA (red in kDNA). The plant pathogenic fungus *Fusarium graminearum* shows A-to-I editing of nuclear transcripts during the late sexual phase. Editing leads to diverse changes at the protein level as shown in the blue-outlined box. Editing of distinct transcripts may affect the maturation of fruiting bodies and the formation and discharge of ascospores (blue arrows), which are the primary inoculum of this fungus. A, adenosine; I, inosine, kDNA, kinetoplastid DNA.

mRNA editing is the occurrence of base substitutions and short insertions or deletions (indels) in an mRNA that could alternatively be directly encoded by the genomic DNA [2]. Typical mRNA editing events are uridine (U) indels as well as cytosine (C)-to-U deamination, reverse U-to-C editing, and adenosine (A) to inosine (I) deamination (Fig 1). Effectively, A-to-I editing generates A-to-guanosine (G) substitutions in coding RNA, because the ribosome interprets I as G during translation.

Many land plant lineages show extensive C-to-U editing and sometimes U-to-C editing in mitochondrial and plastid mRNA, a process that seems to be independent of transcription [5,6,7]. Similar editing events as well as mechanistically distinct U indels and C insertions occur in mitochondria of some metazoan species, trypanosomes, and myxomycetes, among others [2,8]. A-to-I editing of nuclear protein-coding transcripts has been described for some metazoa and filamentous fungi [2,9]. Editing of organellar and nuclear mRNA have opposing effects on proteins. In general, editing of organellar transcripts is restorative, whereas editing of nuclear transcripts leads to proteome diversification [8,10; Fig 1]. The following sections will focus on A-to-I editing of nuclear mRNA.

## Q2: Is mRNA editing common in fungi?

The occurrence of mRNA editing in fungi was revealed only recently for the basidiomycetes *Ganoderma lucidum* and *Pleurotus ostreatus* as well as for the filamentous ascomycetes *F*. *graminearum*, *F*. *verticillioides*, *Neurospora crassa*, *N*. *tetrasperma*, *Pyronema confluens*, and *Sordaria macrospora* [11,12,13,14,15]. In the basidiomycete *G*. *lucidum*, editing shows neither a base change nor a tissue preference [14]. However, editing in ascomycetes shows a preference for A-to-I RNA editing specifically during fruiting body formation [11,12,13]. In *F*. *graminearum* and *N*. *crassa*, editing was detected only in datasets from sexually developing samples, not from asexual spores or vegetative mycelia [11,13]. Interestingly, the ascomycetous yeast *Schizosaccharomyces pombe* does not show evidence of mRNA editing during meiosis [12]. Thus, RNA editing seems to be restricted to multicellular fungi—specifically A-to-I RNA editing to filamentous ascomycetes that generate fruiting bodies.

## Q3: How is RNA editing catalyzed?

Metazoan A-to-I RNA editing of nuclear transcripts is catalyzed by adenosine deaminases acting on RNA (ADARs) [16]. These enzymes deaminate the adenine base to hypoxanthine, resulting in an I instead of an A nucleotide. ADARs contain a deaminase domain and dsRNA-binding domains that bind to double-stranded RNA regions in which the editing sites are located [17]. Besides RNA secondary structure, the base opposite to the target A (preferentially a C) and the flanking nucleotides affect editing efficiency. The human genome encodes five ADARs, two of which show editing activity.

Fungi, like plants, do not encode ADAR homologs, and it remains unknown how fungi catalyze A-to-I RNA editing [9,17]. Fungal editing, in contrast to metazoan editing, preferentially targets As in hairpin loops, and loop stability affects editing efficiency [11]. Furthermore, the sequence context of the editing site differs from that described for human ADARs. Liu and colleagues [11] suggested that adenosine deaminases acting on tRNA (ADATs) may mediate mRNA editing in fungi, possibly together with specific cofactors. Indeed, ADATs were recently shown to catalyze the deamination of adenosines in both tRNA and mRNA in bacteria [18]. However, deletion of the *F*. *graminearum* ADAT-encoding gene *FgTAD1* had no effect on editing, and deletion of *FgTAD2* and *FgTAD3* may be lethal [11]. Thus, the enzymatic activity determining fungal A-to-I RNA editing remains obscure.

## Q4: What are the consequences of RNA editing?

As mentioned above, A-to-I editing of nuclear transcripts leads to proteome diversification. A-to-I editing in humans occurs in a tissue-specific fashion and targets mostly noncoding regions. The few targeted protein-coding transcripts are related to neurological functions [19].

A-to-I editing in filamentous ascomycetes is similarly restricted to a specific stage in the fungal life cycle (see Q2). However, in contrast to metazoan editing sites, most fungal A-to-I editing sites are located in coding regions [11,12,13]. Editing thus can result in synonymous and nonsynonymous codon changes, stop codon loss, and premature stop codon correction in pseudogenes [11,12,13; Fig 1]. In the latter case, editing affects stop codons in in silico wrongly annotated introns that were automatically annotated to avoid the stop codon(s) and keep conserved downstream sequences in the gene model. Thus, editing leads to synthesis of a full-length protein, as described for the *F*. *graminearum* PUK1 kinase [11].

The question whether editing is adaptive or nonadaptive is still under debate. RNA editing allows the generation of divers proteins from one gene, and it has thus been hypothesized that it may facilitate adaptive evolution [20]. In cephalopods, behavioral complexity has been correlated with extensive editing of neuronal transcripts, which is under positive selection [21]. Editing sites introducing nonsynonymous codon changes were found to be highly adaptive and under positive selection in *N*. *crassa* [13]. However, in humans, only a few codon-changing editing events have been associated with altered protein functions, and it has been proposed that most recoding editing events occur as a result of promiscuous editing by ADARs [22]. A neutral evolution model as proposed by, e.g., Gray [23], might also explain the little conservation of individual editing sites, whereas over 20,000 editing sites were found in each *F*. *graminearum*, *N*. *crassa*, and *N*. *tetrasperma*—only 454 are conserved between all three species [13].

## Q5: How does RNA editing relate to fungal pathogenesis?

A correlation of RNA editing and pathogenesis has long been known from trypanosomes, e.g., vertebrate parasites like *T*. *brucei* and *T*. *cruzi*, causing sleeping sickness and Chagas disease in humans, respectively [24]. The first editing event was detected because the trypanosomal mitochondrial *coxII* gene does contain frame-shifts, implying a faulty gene sequence that needs to be corrected for proper biological function of the encoded protein [25]. The single mitochondrion of *T*. *brucei* displays an adaptation to the parasite lifestyle; the procyclic form in the tsetse fly has a standard mitochondrion, whereas the slender bloodstream form (BF) has a tubular mitochondrion with a nonfunctional respiratory chain [26]. Editing activity per se is essential for both the procyclic and the BF type of *T*. *brucei* [27]. However, editing of distinct transcripts may be essential just in the BF form [26,28,29].

Recently, mRNA editing by ADAT activity was shown to occur in *Escherichia coli*. There, editing targets evolutionary conserved toxin—antitoxin pairs. Editing of the *hokB* toxin transcript increased toxicity and was conserved in the pathogenic bacteria *Klebsiella pneumoniae* and *Yersinia enterocolitica* [18], revealing another correlation of editing and pathogenicity.

Fungal nuclear genes whose transcripts are affected by editing tend to have a role in late sexual development, i.e., meiotic spore (ascospore) formation and/or ascospore discharge [11,15,30; Fig 1]. This observation is of interest because ascospores are the primary inoculum of several phytopathogenic ascomycetes, including the wheat and barley pathogen *F*. *graminearum*, *Sclerotinia sclerotiorum*, causing stem rot, or *Blumeria graminis* f. sp. *tritici*, causing wheat powdery mildew [31,32,33; Fig 1]. Whether A-to-I mRNA editing is essential for ascospore generation remains to be determined, like the enzymatic activity underlying fungal editing. Ultimately, however, one may envision a fungal-specific editing factor as a drug target to control those phytopathogenic fungi that use ascospores as primary infecting agents.
